# Supplementary material for: Generative prediction of causal gene sets responsible for complex traits
Source: Proc Natl Acad Sci U S A. 2025 Jun 12;122(24):e2415071122. doi: 10.1073/pnas.2415071122 (PMC12184495; doi:10.1073/pnas.2415071122)
Supplement: Supplementary file 1 — Appendix 01 (PDF) [file pnas.2415071122.sapp.pdf]

Supporting Information for

Generative prediction of causal gene sets responsible for complex traits

Benjamin Kuznets-Speck, Buduka K. Ogonor, Thomas P. Wytock, and Adilson E. Motter

Corresponding author: Adilson E. Motter
E-mail: motter@northwestern.edu

This PDF file includes:

Supporting Information Text
Figs. S1 to S5
Tables S1 to S6
Caption for Dataset S1
References for SI citations

Other supplementary materials for this manuscript include the following:

Dataset S1

Supporting Information Text

This Supporting Information file contains a detailed discussion of the choice of the Euclidean metric underlying our optimization, figures that support the analysis presented in the main text, an extended description of the genes associated with changes in trait phenotypes beyond those discussed in the main text, and figures analyzing the results for the pancancer, MODY3, and inflammatory bowel disease (IBD) traits. The text contains a detailed discussion of the genes associated with each trait in Table 1 that is not explicitly discussed in the main text, with the corresponding genes laid out in Tables S1-S6. Figs. S1 and S2 contain numerical and statistical analysis relevant to the identification of causal genes and significant gene pairs, respectively. The results for co-occurring genes in pancancer metastasis are contained in Fig. S3A,B, and the corresponding results for IBD are in Fig. S3C,D. Figs. S4-S5 concern a comparison of our method with existing ones in the cases of MODY3 and IBD. The first trait phenotype is purported to be caused by a mutation to a single gene, and in Fig. S4 we show that the genes identified by our method overlap with the known downstream effects of that mutation. IBD contains over 1,000 TWAS-associated genes, 44 of which overlap with our perturbations. In Fig. S5, we show that the occurrence frequency of a perturbation is associated with the gene being identified by TWAS at a much higher rate than for differentially expressed genes.

Justification of the Euclidean metric

In this section, we motivate why we choose to formulate optimization in Eqs. [1-2] and [4] of the main text in terms of a Euclidean distance between the baseline and variant states. We recall that the eigengenes are selected for causality via the fine-mapping procedure, and thus the Euclidean norm posits that any selected eigengene could equally lead to a change in phenotype. We comment on four representative alternative choices for the distance:

(1) Minkowski distances, which allow for p—the polynomial order of the norm—to be different from 2 (the Euclidean case);

(2) scaled Euclidean metrics, which allow for the different dimensions to be differently weighted;

(3) similarity metrics, such as the cosine distance, which characterize the alignment between state vectors; and

(4) arbitrary curvilinear metrics, which are well-suited for measuring distance in curved spaces.

Type (1) notions of distance satisfy the triangle inequality when $p \geq 1$, so we focus on those cases. When $p = 1$, the decrease in the objective function is piecewise constant, and the optimal points occur at cusps, but the location of optimal point remains the same. Thus, the perturbations identified by the method presented in the paper is expected to yield similar results, though the value of the regularization parameter λ could change. At the same time, the optimization would likely be more expensive due to the large number of non-differentiable points. For $p > 1$, the objective function is smooth, but the optimal point is again the same, so we expect the selected genes to remain largely similar for other values of p, although some specifics may change in the process of optimization.

Type (2) notions of distance can readily incorporate the existing data and/or trait-specific biological knowledge to reweight the causal eigengenes. One natural weighting is to rescale the projection along each causal eigengene by the (dataset-wide) standard deviation of expression across that same eigengene. This choice would remove any extensive effects of the selected eigengenes (i.e., eigengenes with more weight among highly-expressed genes would be treated on equal footing with lower-expressed genes). In general, reweighting implicitly reflects a choice that the absolute change in expression of certain genes is more important to the phenotype than others. To remain unbiased as to which absolute changes in gene expression influence phenotype, we did not employ such a reweighting here.

Type (3) notions of distance concern only the direction, but not the magnitude of the changes. However, as far as cell behavior is concerned, both the magnitude and direction of the change are expected to be important. We explicitly account for this by restricting the range of perturbation strengths we allow in Eqs. [1-2] and [4] of the main text, and we find cases in which multiple perturbations that point in almost the same direction in transcriptional space are needed to realize a change in the cell behavior.

Type (4) notions of distance require the specification of a global metric tensor at all points in transcriptional space, which implicitly makes substantial assumptions about the transcriptional regulatory network that are difficult to verify with the existing data. As a result, we eschewed implementing metrics of this type in Eqs. [1-2] and [4] of the main text.

Speaking loosely, the Euclidean metric is a parsimonious choice that treats the causal eigengenes on equal footing.

Genes implicated in complex traits

We now discuss the remaining complex traits mentioned in Table 1 but not otherwise discussed in detail in the main text, and the gene perturbations our method associates with their phenotypes. We recall that we use italicized capital letters to refer to genes, while unitalicized capital letters are used for acronyms denoting traits.

Inflammatory bowel disease. IBD, which includes ulcerative colitis and Crohn's disease, is a chronic intestinal inflammatory disease associated with immune system dysregulation in which an imbalance occurs between anti-inflammatory and pro-inflammatory responses (S1). Accordingly, some of the perturbations that account for the largest transcriptional differences between the average healthy transcriptional profile and the average IBD-associated transcriptional profile are associated with genes involved in immune system function (see Table S1).

Several of the genes in Table S1 have also been found in the literature to be differentially expressed in inflammatory tissues. These genes include:

(1) *CPSF3*, which has been found to be markedly higher in ulcerative colitis tissues than in healthy tissues (S1);

(2) *CD86*, which is upregulated in B cells of mouse models and human patients with IBD (S2);

(3) *PROX1*, which is upregulated in Crohn's disease patients (S3); and

(4) *CDK12*, which is often downregulated to mitigate inflammatory disease (S4).

All of these genes make sense biologically since they are known to have roles in regulating immune function.

Food allergy. Food allergies are a sustained overreaction of the immune system to harmless environmental factors, with disruptions in early immune development likely playing a role (S5, S6). Our method implicates several genes crucial to immune system development and immune response modulation, including *CEBPA, LEF1, ERG, TCF7L1, ONECUT2, ETV1*, and *CBFB* (see Table S2). Further, *LEF1* coexpression with *TCF1* (which is in the same protein family as *TCF7L1*) has been shown to lead to chronic activation of T-helper cells, likely due to their combined roles in the Wnt pathway critical for T-cell memory (S5).

Cancer metastasis. Cancer cells undergo a series of transcriptomic and physiological changes that give rise to metastases in different tissues, with the cells' phenotypic plasticity playing a role (S7). Consistent with this observation, we find that genes related to countervailing processes of the epithelial-mesenchymal transition (EMT) and tumor suppression are implicated in cancer metastasis by our method (Table S3). For example, our method identifies *PTHLH*, which is associated with cell migration (S8). In other cases, our method identifies related genes whose polarity of perturbation (up or down regulation) runs counter to that observed in previous experiments. Such genes include *FAM83H-AS1*, also associated with cell migration (S9), and *MYCN*, which is associated with tumor proliferation (S10). In these instances, the same cellular processes are targeted, but outside factors may alter the way that the specific gene upregulations and downregulations influence phenotype.

Age-related macular degeneration. Age-related macular degeneration (AMD) is a leading cause of vision impairment and loss (S11). Disease progression is characterized by the build-up of extracellular deposits in the macula (an anatomical structure within the retina of the eye) and the ensuing degeneration of photoreceptors and nearby tissue (S12). Dysregulation of lipid, vascular, inflammatory, and extracellular matrix pathways have been implicated in this disease (S11). Accordingly, several of the perturbations for AMD identified in Table S4 are in genes involved in these pathways. Specifically, *MIR29A* was found to disrupt the formation of blood vessels in the eye (S13), while a polymorphism in *IGF1R* (a gene with a role in inflammatory responses and angiogenesis) is significantly associated with advanced AMD (S14).

Type 1 diabetes. Type 1 diabetes (T1D) progresses through autoimmune attacks on insulin-producing beta cells within the pancreas (S15). In agreement with this, our method identifies perturbations to genes involved in immune development and response, like *MIR126, CEBPA*, and *ZAP70*, as causal in T1D. Interestingly, we also find perturbations to genes with functions relevant to the pathogenesis of type 2 diabetes (T2D), including insulin resistance and storage and metabolic breakdown of fats (S16). We further find perturbations to genes involved in vascularization, as in processes known to be disrupted as part of the long-term complications of T2D (S16).

Non-small cell lung cancer. Non-small cell lung cancer (NSCLC) represents 85% of new lung cancer cases (S17). Our predicted perturbations involve several genes associated with cell growth, proliferation, and tumor suppression. These include *EGFR*, which is expressed in a variety of human tumors, including NSCLC (S18). Furthermore, *BCL11B* has been shown to have tumor suppressing functionality (S19).

Supporting Information Figures

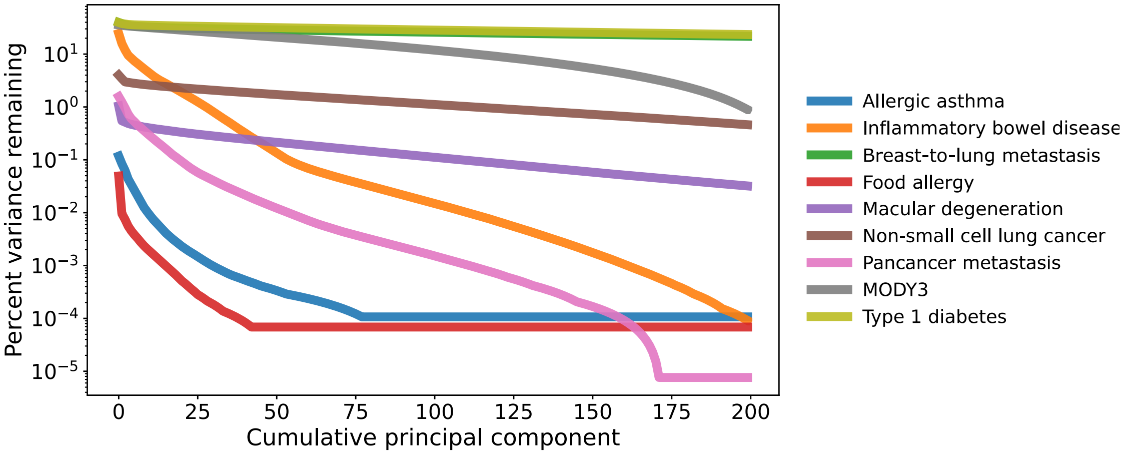

Fig. S1. Justification for using $d = 200$ eigengenes. The percent of unexplained variance in the data is plotted as a function of the number of principal components (ordered by the fraction of variance explained). The top 200 eigengenes explain $> 99\%$ variance for all traits, except cancer metastasis and type 1 diabetes, where $> 77\%$ of the variance is explained.

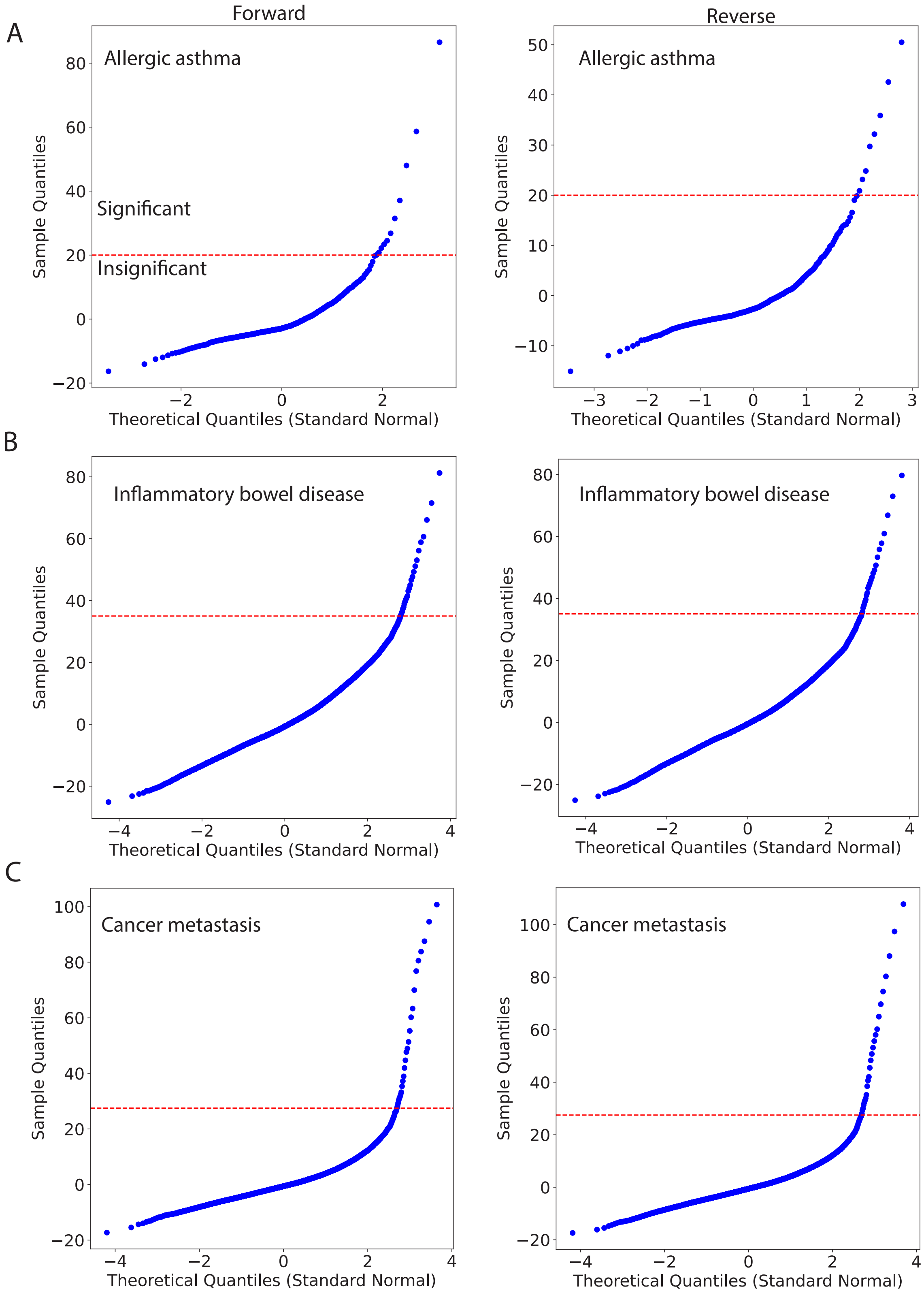

Fig. S2. Quantile-quantile plots for allergic asthma (*A*), inflammatory bowel disease (*B*) and pancancer metastasis (*C*). Horizontal lines indicate thresholds above which co-occurring gene pairs are considered significant. Each case shows the baseline-to-variant (*Left*) and reverse (*Right*) directions.

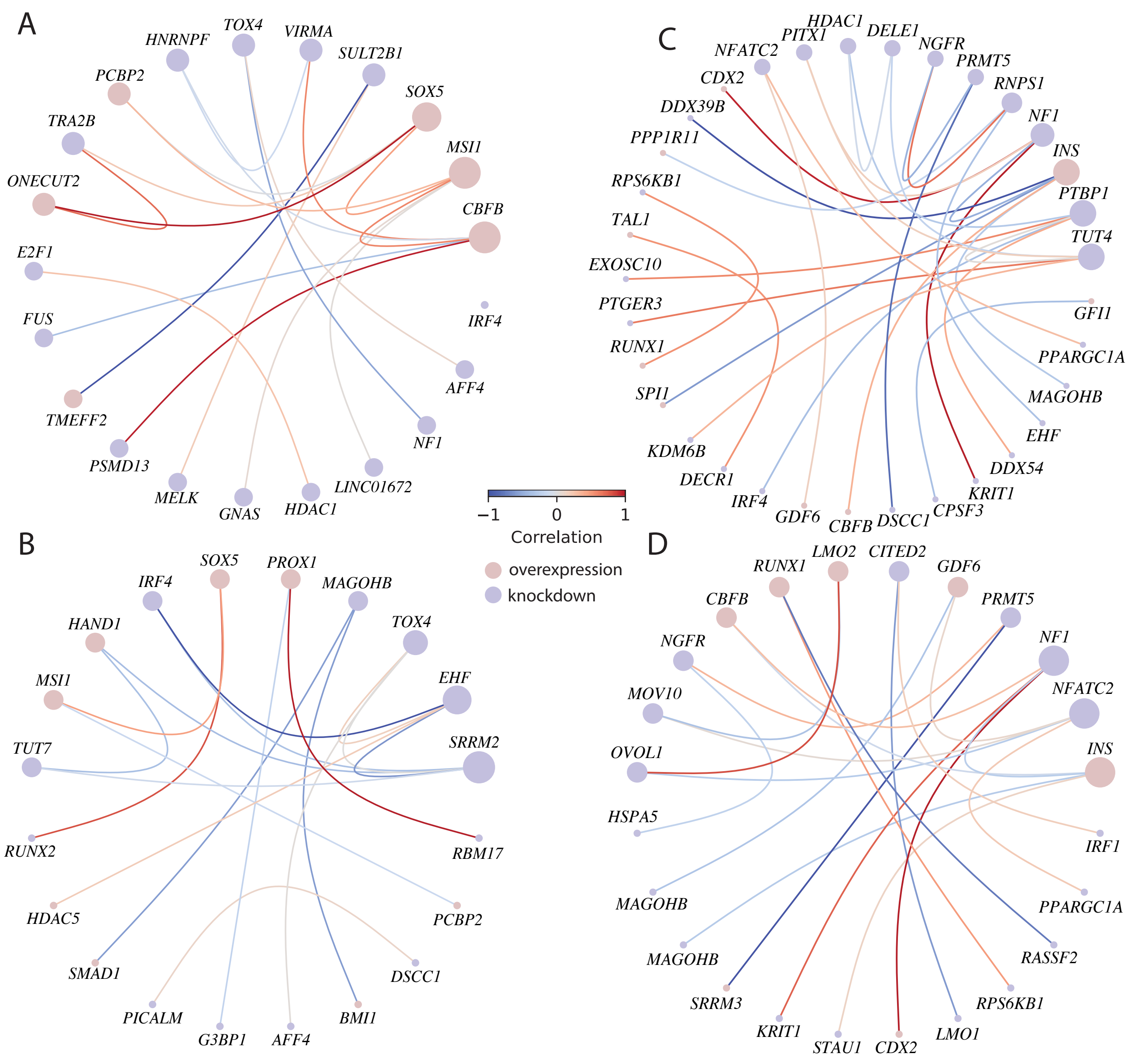

Fig. S3. Gene pair co-occurrence networks for (*A*, *B*) pancancer metastasis and (*C*, *D*) inflammatory bowel disease. The baseline-to-variant pairs are indicated in (A, C) and reverse pairs in (B, D). The edge colors indicate correlations between the genes, the node colors indicate the type of perturbation, and the node sizes are proportional to the number of edges.

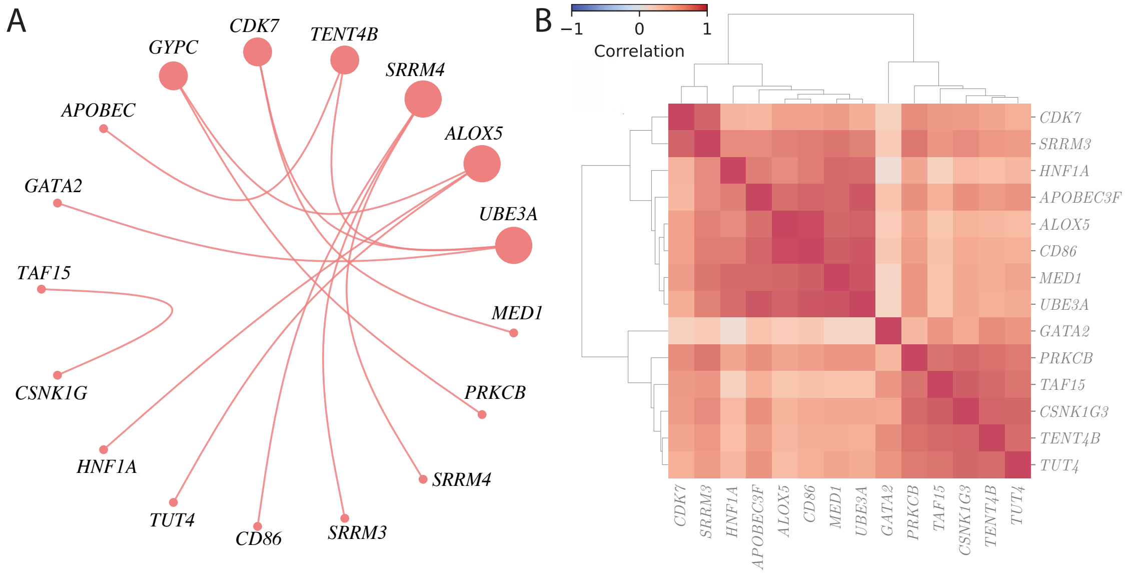

Fig. S4. Co-occurring genes in the MODY3 trait and the correlations between them. (*A*) Gene co-occurrence network in the baseline-to-variant direction. (*B*) Color-coded gene expression correlations of the genes in (A) across all transcriptional responses in the library. The transcriptional responses to *SRRM4* and *GYPC* appear in the library but their expressions are not measured in the dataset, so they do not appear in (B).

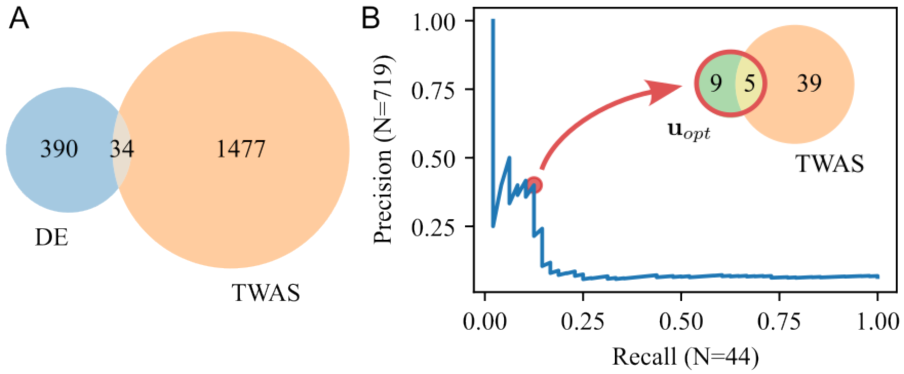

Fig. S5. Comparison of genes identified by differential expression, u_{opt} (our method), and TWAS for inflammatory bowel disease. (*A*) Venn diagram of the differentially expressed genes in GSE193677 (S20) and all TWAS genes associated with inflammatory bowel disease. Only 8% of the differentially expressed (DE) genes are also implicated by TWAS. (*B*) Precision-recall curve of the 719 unique genes associated with columns of the perturbation matrix B versus the 44 TWAS genes that overlap with these perturbed genes. Genes were ranked by the number of times they appear in the point-to-point forward optimizations for IBD. For the top n genes, the precision is the fraction of the n genes that are in the set of TWAS genes, while the recall is the fraction of the 44 TWAS genes contained in the top n genes. The red dot indicates $n = 14$ genes of which 5 overlap with TWAS, as indicated in the Venn diagram.

Supporting Information Tables

Table S1. Transcriptional perturbations associated with inflammatory bowel disease.

Gene	Annotation
CPSF3$^-$	post-transcriptional processing (S21)
UBE2O$^-$	protein quality control, blood cell differentiation (S22)
CD86$^-$	T-cell activation (S23)
PROX1$^+$	developmental transcription factor (S3)
CDK12$^-$	cell cycle progression (S24)
ADARB1$^-$	adenosine-to-inosine (A-to-I) RNA editing (S25)
RBJ$^-$	GTPase signaling protein (S26)
SLIRP$^-$	RNA binding protein (S27)
NOTCH1$^+$	intracellular signaling, cell fate (S28)
SOX2$^-$	embryonic development (S29)
RNF43$^+$	DnaJ heat shock, regulation of Wnt signaling, tumor suppression protein (S30)
NAB2$^+$	transcriptional corepressor of EGR genes (S31)

Table S2. Transcriptional perturbations associated with food allergy.

Gene	Annotation
CEBPA$^+$	transcription factor, roles in adipogenesis, immune cell differentiation, metabolic homeostasis (S32, S33)
LINC00673$^-$	expression regulation, tumor suppression, involved in cancer progression (S34)
LEF1$^+$	transcription factor in the Wnt/β-catenin signaling pathway, hematopoiesis, immune response modulation (S5, S35)
ERG$^+$	transcription factor in the ETS family, with roles in immune cell differentiation (S36)
ADARB1$^-$	adenosine-to-inosine (A-to-I) RNA editing (S25)
TCF7L1$^-$	regulator in the Wnt/β-catenin signaling pathway–roles in T-cell differentiation, epithelial barrier function, and immune response (S37)
ONECUT2$^-$	epithelial differentiation, immune cell regulation, and tissue development (S38)
RBPJ$^-$	transcriptional mediator of Notch signaling–differentiation, inflammation, and tissue homeostasis (S39)
ETV1$^-$	transcription factor from ETS family, known for role in cell differentiation, immune regulation, and tissue homeostasis (S40)
MSI1$^+$	RNA-binding protein, with roles in stem cell maintenance, epithelial regeneration, and immune cell function (S41)
CBFB$^-$	regulates immune cell development, osteogenesis, hematopoiesis (S42)
BMPR2$^-$	receptor in the TGF-β/BMP signaling pathway, which regulates immune responses, tissue homeostasis, and inflammation (S43)

Table S3. Transcriptional perturbations associated with cancer metastasis to the lung.

Gene	Annotation
LPAR2$^-$	G-protein-coupled receptor, involved cell proliferation, migration, and survival (S44)
FAM83H-AS1$^-$	gene regulation via mRNA stabilization, involved in cell proliferation and migration (S9)
MYCN$^-$	gene regulation and embryonic development, cell proliferation (S45)
EZH2$^+$	epigenetic regulation through histone methylation, neural development (S46)
RNF43$^+$	DnaJ heat shock, regulation of Wnt signaling, tumor suppression (S30)
JUNB$^-$	transcription factor, cell differentiation, immune regulation, tumorigenesis (S47)
HOXC6$^-$	transcription factor involved in embryonic development and cell differentiation, contributes to tumor progression and metastasis (S48)
PPP1R11$^+$	regulatory inhibitor, cellular homeostasis, modulates immune response (S49)
BPIFB3$^-$	regulation of autophagy and endoplasmic reticulum (ER) morphology (S50)
PTHLH$^+$	calcium homeostasis and cellular proliferation (S51)
VIRMA$^-$	regulates immune cell development, epithelial integrity, and inflammation (S52)
PCBP2$^-$	RNA binding protein (S53)

Table S4. Transcriptional perturbations associated with age-related macular degeneration.

Gene	Annotation
MAPK3$^+$	cell signaling, growth, differentiation, and development (S54)
EZH1$^-$	epigenetic regulation through histone methylation, neural development (S46)
HNF1A$^+$	transcription factor regulating metabolic processes (S55)
BRD4$^-$	scaffold protein for transcription factors, inflammatory response (S56)
UBE3A$^-$	protein degradation, neuronal development, lipid metabolism (S57)
STK11$^-$	growth regulation, tumor suppression (S58)
NDP$^-$	Wnt/β-catenin signaling pathway, retinal vascular development (S59)
NEAT1$^-$	immune regulation, nuclear sequestration, neural homeostasis / stress response (S60)
G3BP1$^-$	stress response, mRNA regulation via sequestration and degeneration (S61)
MIR29A$^-$	regulation of extracellular matrix genes, angiogenesis (S13)
IGF1R$^+$	development, cell growth, metabolic regulation (S62)
ADAR$^-$	adenosine-to-inosine (A-to-I) RNA editing (S25, S63)

Table S5. Transcriptional perturbations associated with type 1 diabetes.

Gene	Annotation
COP1$^-$	insulin secretion, protein degradation, tumor suppression (S64)
UBE3A$^-$	protein degradation, neuronal development, lipid metabolism (S57)
PHF8$^+$	epigenetic regulation, neuronal development (S65)
RNF20$^-$	epigenetic regulation, adipogenesis (S66)
MIR126$^+$	epigenetic regulation of metabolism, inflammatory response, angiogenesis (S67)
CEBPA$^+$	transcription factor, roles in adipogenesis, immune cell differentiation, metabolic homeostasis (S32, S33)
C9orf72$^-$	metabolic homeostasis, motor neuron function (S68)
BMPR2$^-$	osteogenesis, cell differentiation, vascular function (S43)
MOV10$^-$	gene regulation, development (S69)
MIR29B1$^-$	regulation of extracellular matrix genes, promotion of endothelial function (S70, S71)
ZAP70$^-$	immune regulation and development (S72)
BMI1$^+$	transcriptional repressor, mediation of signaling pathways in cardiovascular tissues (S73)

Table S6. Transcriptional perturbations associated with non-small cell lung cancer.

Gene	Annotation
EGFR$^-$	cell growth and proliferation (S74)
KRTAP10-6$^-$	hair follicle structure (S75, S76)
BPIFB6$^-$	secretory pathway regulator (S77)
BCL11B$^-$	immune and neuronal development, tumor suppression (S78)
JUNB$^-$	cell differentiation, immune regulation, tumorigenesis (S47)
MYC$^-$	growth regulation (S79)
TP63$^-$	epithelial differentiation (S80)
CELSR2$^-$	neuronal development, axon growth (S81)
OVOL1$^-$	mesenchymal to epithelial transition (S82)
STK11$^-$	growth regulation, tumor suppression (S58)
U2SURP$^-$	pre-mRNA splicing (S83)
NR2F2$^-$	development, cell differentiation (S84)

Supporting Information Datasets

Dataset S1 **(Dataset_S1-transcriptional_response_metadata.xlsx)**
Excel file of metadata including the SRA accession numbers of the publicly available data used to specify the transcriptional response matrix B. The NCBI Bioproject, cell line, cell type, treatment, and genotype of the initial (unperturbed) and treated (perturbed) states are recorded (left to right) in this table.

References

[S1] Y Chen, J Lei, S He, m6A modification mediates mucosal immune microenvironment and therapeutic response in inflammatory bowel disease. *Front. Cell Dev. Biol.* **9**, 692160 (2021).
[S2] I Gadjalova, et al., B cell-mediated CD4 T-cell costimulation via CD86 exacerbates pro-inflammatory cytokine production during autoimmune intestinal inflammation. *Mucosal Immunol.* **17**, 67 (2024).
[S3] W Shen, et al., Decreased expression of Prox1 is associated with postoperative recurrence in Crohn's disease. *J. Crohns Colitis* **12**, 1210 (2018).
[S4] KL Henry, et al., CDK12-mediated transcriptional regulation of noncanonical NF-κB components is essential for signaling. *Sci. Signal.* **11**, eaam8216 (2018).
[S5] R Kratchmarov, et al., TCF1–LEF1 co-expression identifies a multipotent progenitor cell (TH2-MPP) across human allergic diseases. *Nat. Immunol.* **25**, 902 (2024).
[S6] D Martino, et al., Epigenetic dysregulation of naive CD4+ T-cell activation genes in childhood food allergy. *Nat. Commun.* **9**, 3308 (2018).
[S7] B Hamelin, et al., Single-cell analysis reveals inter- and intratumour heterogeneity in metastatic breast cancer. *J Mammary Gland Biol. Neoplasia* **28**, 26 (2023).
[S8] J Urosevic, et al., Colon cancer cells colonize the lung from established liver metastases through p38 MAPK signalling and PTHLH. *Nat. Cell Biol.* **16**, 685 (2014).
[S9] J Zhang, et al., Overexpression of FAM83H-AS1 indicates poor patient survival and knockdown impairs cell proliferation and invasion via MET/EGFR signaling in lung cancer. *Sci. Rep.* **7**, 42819 (2017).
[S10] LA Goodman, et al., Modulation of N-myc expression alters the invasiveness of neuroblastoma. *Clin. Exp. Metastasis* **15**, 130 (1997).
[S11] P Mitchell, G Liew, B Gopinath, TY Wong, Age-related macular degeneration. *Lancet* **392**, 1147 (2018).
[S12] M Fleckenstein, et al., Age-related macular degeneration. *Nat. Rev. Dis. Primers* **7**, 31 (2021).
[S13] D Peng, et al., Anti-angiogenic properties of microRNA-29a in preclinical ocular models. *Proc. Natl. Acad. Sci. U.S.A.* **145**, e2204795119 (2022).
[S14] CJ Chiu, et al., Associations between genetic polymorphisms of insulin-like growth factor axis genes and risk for age-related macular degeneration. *Invest. Ophthalmol. Vis. Sci.* **52**, 9099 (2011).
[S15] PS Linsley, et al., Germline-like TCR-α chains shared between autoreactive T cells in blood and pancreas. *Nat. Commun.* **15**, 4971 (2024).
[S16] E Ahmad, S Lim, R Lamptey, DR Webb, MJ Davies, Type 2 diabetes. *Lancet* **400**, 1803 (2022).
[S17] C Gridelli, et al., Non-small-cell lung cancer. *Nat. Rev. Dis. Primers* **1**, 1 (2015).
[S18] F Hirsch, M Varella-Garcia, F Cappuzzo, Predictive value of EGFR and HER2 overexpression in advanced non-small-cell lung cancer. *Oncogene* **28**, S32 (2009).
[S19] K Kamimura, et al., Haploinsufficiency of Bcl11b for suppression of lymphomagenesis and thymocyte development. *Biochem. Biophys. Res. Co.* **355**, 538 (2007).
[S20] C Argmann, et al., Biopsy and blood-based molecular biomarker of inflammation in IBD. *Gut* **72**, 1271 – 1287 (2023).
[S21] P Shen, et al., Therapeutic targeting of CPSF3-dependent transcriptional termination in ovarian cancer. *Sci. Adv.* **9**, eadj0123 (2023).
[S22] AT Nguyen, et al., UBE2O remodels the proteome during terminal erythroid differentiation. *Science* **357**, eaan0218 (2017).
[S23] P Jeannin, et al., Human Effector Memory T Cells Express CD86: A Functional Role in Naive T Cell Priming. *J. Immunol.* **162**, 2044 (1999).
[S24] V Sundar, et al., Transcriptional cyclin-dependent kinases as the mediators of inflammation-a review. *Gene* **769**, 145200 (2021).
[S25] W Slotkin, K Nishikura, Adenosine-to-inosine RNA editing and human disease. *Genome Med.* **5**, 1 (2013).
[S26] Z Gao, et al., Crystal structure and function of Rbj: A constitutively GTP-bound small G protein with an extra DnaJ domain. *Protein Cell* **10**, 760 (2019).
[S27] TCP Pham, et al., The mitochondrial mRNA-stabilizing protein SLIRP regulates skeletal muscle mitochondrial structure and respiration by exercise-recoverable mechanisms. *Nat. Commun.* **15**, 9826 (2024).
[S28] C Fazio, L Ricciardiello, Inflammation and Notch signaling: a crosstalk with opposite effects on tumorigenesis. *Cell Death Dis.* **7**, e2515 (2016).
[S29] P Xia, et al., Sox2 functions as a sequence-specific DNA sensor in neutrophils to initiate innate immunity against microbial infection. *Nat. Immunol.* **16**, 366 (2015).

[S30] B Holm, S Barsuhn, H Behrens, S Krüger, C Röcken, The tumor biological significance of RNF43 and LRP1B in gastric cancer is complex and context-dependent. *Sci. Rep.* **13**, 3191 (2023).
[S31] M Lucerna, et al., NAB2, a corepressor of EGR-1, inhibits vascular endothelial growth factor-mediated gene induction and angiogenic responses of endothelial cells. *J. Biol. Chem.* **278**, 11433 (2003).
[S32] L Olofsson, et al., CCAAT/Enhancer Binding Protein α (C/EBPα) in Adipose Tissue Regulates Genes in Lipid and Glucose Metabolism and a Genetic Variation in C/EBPα Is Associated with Serum Levels of Triglycerides. *J. Clin. Endocrinol. Metab.* **93**, 4880 (2010).
[S33] R Avellino, R Delwel, Expression and regulation of C/EBPα in normal myelopoiesis and in malignant transformation. *Blood* **129**, 2083 (2017).
[S34] H Guan, et al., Long noncoding RNA LINC00673-v4 promotes aggressiveness of lung adenocarcinoma via activating WNT/β-catenin signaling. *Proc. Natl. Acad. Sci. U.S.A.* **116**, 14019 (2019).
[S35] T Reya, et al., Wnt signaling regulates B lymphocyte proliferation through a LEF-1 dependent mechanism. *Immunity* **13**, 15 (2000).
[S36] AP Ng, et al., An Erg-driven transcriptional program controls B cell lymphopoiesis. *Nat. Commun.* **11**, 3013 (2020).
[S37] YC Wen, et al., TCF7L1 regulates cytokine response and neuroendocrine differentiation of prostate cancer. *Oncogenesis* **10**, 81 (2021).
[S38] MV Luna Velez, et al., ONECUT2 regulates RANKL-dependent enterocyte and microfold cell differentiation in the small intestine; a multi-omics study. *Nucleic Acids Res.* **51**, 1277 (2023).
[S39] M Delacher, et al., Rbpj expression in regulatory T cells is critical for restraining TH2 responses. *Nat. Commun.* **10**, 1621 (2019).
[S40] X Shen, et al., ETV1 positively correlated with immune infiltration and poor clinical prognosis in colorectal cancer. *Front. Immunol.* **13**, 939806 (2022).
[S41] M Forouzanfar, et al., Intracellular functions of RNA-binding protein, Musashi1, in stem and cancer cells. *Stem Cell Res. Ther.* **11**, 193 (2020).
[S42] L Zhao, JL Cannons, LH Castilla, PL Schwartzberg, PP Liu, The Role of CBFβ in T Cell Development. *Blood* **104**, 3234 (2004).
[S43] I Cuthbertson, N Morrell, P Caruso, BMPR2 mutation and metabolic reprogramming in pulmonary arterial hypertension. *Circ. Res.* **132**, 109 (2023).
[S44] S Kuriyama, et al., In vivo collective cell migration requires an LPAR2-dependent increase in tissue fluidity. *J. Cell Biol.* **206**, 113 (2014).
[S45] M Ruiz-Pérez, A Henley, M Arsenian-Henriksson, The MYCN Protein in Health and Disease. *Genes* **8**, 42819 (2017).
[S46] S Lee, et al., The role of EZH1 and EZH2 in development and cancer. *BMB Rep.* **55**, 595 (2022).
[S47] FJ Ren, XY Cai, Y Yao, GY Fang, JunB: a paradigm for Jun family in immune response and cancer. *Front. Cell. Infect. Microbiol.* **13**, 1222265 (2023).
[S48] L Qi, et al., HomeoboxC6 promotes metastasis by orchestrating the DKK1/Wnt/β-catenin axis in right-sided colon cancer. *Cell Death Dis.* **12**, 337 (2021).
[S49] RN Joshi, et al., Phosphatase inhibitor PPP1R11 modulates resistance of human t cells toward Treg-mediated suppression of cytokine expression. *J. Leukoc. Biol.* **106**, 413 (2019).
[S50] A Evans, N Lennemann, C Coyne, BPIFB3 Regulates Endoplasmic Reticulum Morphology To Facilitate Flavivirus Replication. *J Virol* **94** (2020).
[S51] M Yao, et al., Tumor signatures of PTHLH overexpression, high serum calcium, and poor prognosis were observed exclusively in clear cell but not non clear cell renal carcinomas. *Cancer Med.* **3** (2014).
[S52] ZQ Zheng, et al., VIRMA promotes nasopharyngeal carcinoma, tumorigenesis, and metastasis by upregulation of *E2F7* in an m6a-dependent manner. *J. Biol. Chem.* **299**, 104677 (2023).
[S53] W Han, et al., RNA-binding protein PCBP2 modulates glioma growth by regulating *FHL3*. *J. Clin. Invest.* **123**, 2103 (2013).
[S54] P Moustardas, D Aberdam, N Lagali, MAPK Pathways in Ocular Pathophysiology: Potential Therapeutic Drugs and Challenges. *Cells* **12**, 617 (2021).
[S55] K Morita, et al., Common variants of HNF1A gene are associated with diabetic retinopathy and poor glycemic control in normal-weight Japanese subjects with type 2 diabetes mellitus. *JDC* **31**, 483 (2017).
[S56] M Zou, et al., Inhibition of cGAS-STING by JQ1 alleviates oxidative stress-induced retina inflammation and degeneration. *Cell Death Differ.* **29**, 1816 (2022).
[S57] M Loix, N Zelcer, JF Bogie, JJ Hendriks, The ubiquitous role of ubiquitination in lipid metabolism. *Trends Cell Biol,* **34**, 416 (2024).
[S58] D Shackelford, R Shaw, The LKB1–AMPK pathway: metabolism and growth control in tumour suppression. *Nat. Rev. Cancer* **9**, 563 (2009).
[S59] J Wawrzynski, et al., Spectrum of mutations in NDP resulting in ocular disease; a systematic review. *Front. Genet.* **13**, 884722 (2022).
[S60] H An, N Williams, T Shelkovnikova, NEAT1 and paraspeckles in neurodegenerative diseases: A missing lnc found? *Noncoding RNA Res.* **3**, 243 (2018).
[S61] P Sahoo, et al., Disruption of G3BP1 granules promotes mammalian CNS and PNS axon regeneration. *Proc. Natl. Acad.*

Sci. U.S.A. **122**, e2411811122 (2025).
[S62] T Truong, RZ Silkiss, The Role of Insulin-like Growth Factor-1 and Its Receptor in the Eye: A Review and Implications for IGF-1R Inhibition. *Ophthal. Plast. Reconstr. Surg.* **39**, 4 (2023).
[S63] L Yang, et al., Temporal landscape and translational regulation of A-to-I RNA editing in mouse retina development. *BMC Biol.* **22**, 106 (2024).
[S64] R Suriben, et al., β-Cell Insulin Secretion Requires the Ubiquitin Ligase COP1. *Cell* **163**, 1457 (2015).
[S65] H Qi, M Sarkissian, G Hu, Histone H4K20/H3K9 demethylase PHF8 regulates zebrafish brain and craniofacial development. *Nature* **466**, 503 (2010).
[S66] YG Jeon, et al., RNF20 Functions as a Transcriptional Coactivator for PPARγ by Promoting NCoR1 Degradation in Adipocytes. *Diabetes* **69**, 20 (2019).
[S67] A Zampetaki, et al., Plasma microRNA profiling reveals loss of endothelial miR-126 and other microRNAs in type 2 diabetes. *Circ. Res.* **107**, 810 (2010).
[S68] T Wang, et al., C9orf72 regulates energy homeostasis by stabilizing mitochondrial complex I assembly. *Cell Metab.* **33**, 531 (2021).
[S69] A Nawaz, T Shilikbay, G Skariah, S Ceman, Unwinding the roles of RNA helicase MOV10. *Wiley Interdiscip. Rev. RNA* **13**, e1682 (2022).
[S70] J Dooley, et al., The microRNA-29 Family Dictates the Balance Between Homeostatic and Pathological Glucose Handling in Diabetes and Obesity. *Diabetes* **65**, 53 (2015).
[S71] D Jensen, et al., Broad-acting therapeutic effects of miR-29b-chitosan on hypertension and diabetic complications. *Mol. Ther.* **30**, 3462 (2022).
[S72] A Fischer, et al., ZAP70: A master regulator of adaptive immunity. *Semin. Immunopathol.* **32**, 107 (2010).
[S73] D Yang, H Liu, Z Yang, D Fan, Q Tang, BMI1 in the heart: Novel functions beyond tumorigenesis. *eBioMedicine* **63**, 103193 (2021).
[S74] RS Iyer, et al., Drug-resistant *EGFR* mutations promote lung cancer by stabilizing interfaces in ligand-free kinase-active *EGFR* oligomers. *Nat. Commun.* **15**, 2130 (2024).
[S75] T Litman, W Stein, Ancient lineages of the keratin-associated protein (*KRTAP*) genes and their co-option in the evolution of the hair follicle. *BMC Ecol. Evo.* **23** (2023).
[S76] C Hu, et al., A prediction model integrated genomic alterations and immune signatures of tumor immune microenvironment for early recurrence of stage I NSCLC after curative resection. *Transl. Lung Cancer Res.* **11** (2022).
[S77] CC Morosky S, Lennemann NJ, BPIFB6 regulates secretory pathway trafficking and enterovirus replication. *J. Virol.* **90**, 5098 (2016).
[S78] A Gutierrez, et al., The BCL11B tumor suppressor is mutated across the major molecular subtypes of T-cell acute lymphoblastic leukemia. *Blood* **118**, 4169–4173 (2011).
[S79] R Dhanasekaran, et al., The *MYC* oncogene - the grand orchestrator of cancer growth and immune evasion. *Nat. Rev. Clin. Oncol.* **19**, 23 (2022).
[S80] A Yang, et al., *p63*, a *p53* Homolog at 3q27–29, Encodes Multiple Products with Transactivating, Death-Inducing, and Dominant-Negative Activities. *Mol. Cell* **2**, 305 (1998).
[S81] Q Wen, et al., Inactivating *Celsr2* promotes motor axon fasciculation and regeneration in mouse and human. *Brain* **145**, 670 (2022).
[S82] H Roca, et al., Transcription Factors OVOL1 and OVOL2 Induce the Mesenchymal to Epithelial Transition in Human Cancer. *PLoS ONE* **8**, e76773 (2013).
[S83] L Deng, et al., MYC-driven U2SURP regulates alternative splicing of SAT1 to promote triple-negative breast cancer progression. *Cancer Lett.* **560**, 216124 (2023).
[S84] J Yang, W Sun, C G, Roles of the NR2F Family in the Development, Disease, and Cancer of the Lung. *J. Dev. Biol.* **12**, 24 (2024).
